# Assessing ADHD symptoms in children and adults: evaluating the role of objective measures

**DOI:** 10.1186/s12993-018-0143-x

**Published:** 2018-05-18

**Authors:** Theresa S. Emser, Blair A. Johnston, J. Douglas Steele, Sandra Kooij, Lisa Thorell, Hanna Christiansen

**Affiliations:** 1 Clinical Child and Adolescent Psychology, Department of Psychology, Philipps University Marburg, Gutenbergstr. 18, 35037 Marburg, Germany; 2 Clinic for Child and Adolescent Psychiatry, University Clinic Würzburg, Margarete-Höppel-Platz 1, 97080 Würzburg, Germany; 3 Division of Neuroscience, Medical Research Institute, Ninewells Hospital and Medical School, University of Dundee, Dundee, DD1 9SY UK; 4 School of Medicine (Neuroscience), University of Dundee, Dundee, DD1 9SY UK; 5 PsyQ, Psycho-medical Programs, Expertise Center Adult ADHD, Jan van Nassaustraat 125, 2596 BS The Hague, The Netherlands; 6 Department of Clinical Neuroscience, Karolinska Institutet, Tomtebodavägen 18A, 5th floor, 171 77 Stockholm, Sweden

**Keywords:** ADHD, Children/adults, Objective assessment, Classification, Support vector machines

## Abstract

**Background:**

Diagnostic guidelines recommend using a variety of methods to assess and diagnose ADHD. Applying subjective measures always incorporates risks such as informant biases or large differences between ratings obtained from diverse sources. Furthermore, it has been demonstrated that ratings and tests seem to assess somewhat different constructs. The use of objective measures might thus yield valuable information for diagnosing ADHD. This study aims at evaluating the role of objective measures when trying to distinguish between individuals with ADHD and controls. Our sample consisted of children (n = 60) and adults (n = 76) diagnosed with ADHD and matched controls who completed self- and observer ratings as well as objective tasks. Diagnosis was primarily based on clinical interviews. A popular pattern recognition approach, support vector machines, was used to predict the diagnosis.

**Results:**

We observed relatively high accuracy of 79% (adults) and 78% (children) applying solely objective measures. Predicting an ADHD diagnosis using both subjective and objective measures exceeded the accuracy of objective measures for both adults (89.5%) and children (86.7%), with the subjective variables proving to be the most relevant.

**Conclusions:**

We argue that objective measures are more robust against rater bias and errors inherent in subjective measures and may be more replicable. Considering the high accuracy of objective measures only, we found in our study, we think that they should be incorporated in diagnostic procedures for assessing ADHD.

## Background

Attention deficit/hyperactivity disorder (ADHD) is characterized by a combination of age-inappropriate levels of inattention, impulsive behavior and hyperactivity. Symptoms must become apparent before the age of 12 years and cause significant impairments in more than one setting, e.g., at school or work, or with family and peers [1]. The diagnostic and statistical manual of mental disorders (DSM V; [1]) distinguishes three subtypes of ADHD, the predominantly inattentive type (IA), the predominantly hyperactive/impulsive type (HI) and the combined type (C). Children of the IA type show more than six out of the nine relevant symptoms specified as inattentive behavior and less than six out of the nine relevant symptoms specified as hyperactive/impulsive behavior. The predominantly HI type is characterized by more than six HI symptoms and less than six IA symptoms, whereas children of the C type show more than six symptoms in both areas. Although ADHD was long regarded solely as a childhood disorder, it is now agreed that the disorder persists into adulthood (e.g., [2, 3]), and even into old age [4, 5]. Estimations of adulthood ADHD’s prevalence rates range from 2.5 to 5% [6–12], slightly smaller than those reported for children and youths that range from 5.0 to 7.1% [13, 14].

### Assessment of ADHD using ratings and tests

Most diagnostic guidelines (e.g., [15–17]) require that ADHD be assessed and diagnosed by relying on information provided via a variety of methods (e.g., clinical interviews, observations and ratings) and collected from multiple sources (e.g., parents and teachers). However, using subjective measures always incorporates the risk of informant biases [18] and clinicians are often confronted with great inconsistencies between ratings obtained from different sources [19, 20]. Although the discrepancies between different informants can be of clinical relevance [21], the use of objective measures in addition to subjective ratings might yield valuable information facilitating the diagnosis of ADHD. In the present study, we therefore aimed to investigate the role of *objective* measures when trying to distinguish between individuals with ADHD and controls. We also aimed to investigate how objective measures are related to subjective measures by investigating how well we could discriminate between ADHD and controls when using the combination of these two types of measures. The combination of objective and subjective measures may provide additional information than objective measures alone as it has been argued that tests and ratings may capture at least partly different constructs [22, 23] and should not be used interchangeably. Toplak and colleagues [23] argue that one important difference between ratings and tests is that the former measure typical performance (i.e., how an individual normally performs), whereas tests usually capture optimal performance (i.e., how well an individual performs under relatively optimal conditions). Thus, objective measures assess performance free of influences of the different situations. However, this study primarily investigated whether only objective measures would be sufficient to develop a statistical model as the bias and inter-operator error inherent in subjective measures are not well suited to developing a robust and objective classifier. Whilst the value of including subjective measures in a classifier alongside objective measures has been explored, developing an objective statistical method using only subjective data would not be expected to produce a robust classifier that would generalize to corresponding data acquired by other operators.

The relative importance of individual variables towards a diagnosis of ADHD is an issue that has not been empirically examined, at least not in studies employing statistical methods that can handle numerous variables to make an objective prediction. Similarly, few studies focus on objective measurements of ADHD symptom levels rather than constructs (such as executive functioning deficits) that are known to be associated with ADHD.

### Objective measures

#### Test battery of attention

In Germany, where this study was conducted, a frequently used neuropsychological test is the test battery of attention for adolescents and adults (TAP; [24]) or for children aged 6–11 (KiTAP; [25]). The various subtests enable the assessment of aspects of two of the three core symptoms of ADHD, namely inattention and impulsivity. A detailed description of the tasks is provided in the method section. One study using the TAP in a sample of children with ADHD and healthy controls demonstrated that two test measures (reaction time variability of the Go/NoGo task, number of errors of the reaction change task) were needed to classify 90% of the children correctly [26]. Drechsler et al. [27] detected significant group differences between children with and without ADHD in four of the KiTAP’s six subtests. Nevertheless, they did not recommend using it for diagnostic purposes due to its weak specificity. Another study on the psychometric properties of the KiTAP reported values for split-half reliability of .55–.96 for children aged 8–12 [25] and .32–.72 for children aged 6–7 years [28]. The psychometric properties of the TAP/KiTAP are thus not fully satisfactory, and norm references are missing for some age groups. An alternative is the Quantified Behavior Test, a neuropsychological test becoming increasingly important in ADHD diagnostics.

#### The Quantified Behavior Test

The Quantified Behavior Test for children aged 6–12 (QbTest 6–12; [29]) and the Quantified Behavior Test Plus for subjects 12 years and older (Qb+^©^; [30]) are computerized neuropsychological tests that assess the three core symptoms of ADHD using a continuous performance test (CPT). One great advantage of these tests is that in addition to providing estimates of the participant’s performance (e.g., omission and commission errors), they also measure head movements via a motion tracking system. For example, the system generates measures of the time the subject has moved more than 1 cm/s, as well as the distance they traveled during the test or the surface covered through their movements. Reh et al. [31] reported promising results determining the QbTest 6–12’s factorial and discriminant validity with a three-factor solution corresponding to the three areas of ADHD impairment. These explained 76% of the total variance and reliability estimates ranging from α = .60 (impulsivity) to α = .95 (hyperactivity) for these factors. Findings have been less consistent regarding the QbTest 6–12’s convergent and discriminant validity. One study exhibited significant differences between children with ADHD, their siblings, and healthy controls, and the authors identified the factor of hyperactivity as a possible “intermediate phenotype” [32]. Hult et al. [33] examined the diagnostic validity of the QbTest 6–12 applying ROC curves in a clinical sample of children diagnosed with ADHD and a clinical control group of individuals with primarily autism spectrum disorder, observing moderate sensitivity (47–67%) and specificity values (72–84%). In a third study, multi-trait, multi-method analyses comparing self- and observer ratings (Conners 3 rating scales) with objective measures provided support for the convergent validity of the QbTest 6–12 especially for the variables assessing inattention, but discriminant validity was not supported [34]. However, discrimination analyses based on the QbTest 6–12 also achieved 73.8% accuracy in predicting whether a child had an ADHD diagnosis (all subtypes) or not with the variables measuring activity revealing the greatest impact. There are studies of the Qb+^©^, the version used for adolescents and adults, demonstrating high sensitivity (86%) and specificity (83%) when trying to differentiate between subjects with and without ADHD [35, 36]. However, sensitivity dropped substantially when trying to differentiate between individuals with ADHD and other clinical groups such as bipolar II disorder (36%) or borderline personality disorder (41%; [35]). However, in another study, with a large sample of patients that came in for ADHD assessment, we were able to differentiate patients for which an ADHD diagnosis was confirmed (66% of 773 subjects) versus patients that had symptoms of inattention, impulsivity or hyperactivity due to other disorders (34% of 773 subjects). All individuals performed the QbTest, the objective measure also used in this study. Of those individuals predicted not to have an ADHD diagnosis based on the QbTest, 67% actually had no diagnosis; of those individuals predicted to have an ADHD diagnosis, 79% actually had a diagnosis. In the whole sample, the correct classification rate was 76.4%, sensitivity was 90%, and specificity was just 45% [37]. Another study reported satisfactory overall classification rates (87.8% correctly identified ADHD patients), but lower correct prediction rates regarding the area under the curve (AUC) range for sensitivity (36.5–58.5%) and specificity (80–100%; [38]). Hirsch and Christiansen [37] verified the three factorial structure of the Qb+^©^ and provided support for convergent validity using multi-trait, multi-method analyses, but the discriminant validity of this instrument was only partially supported. The measure of impulsivity has been shown to be the least sensitive symptom with regard to discriminating between adults with and without ADHD as well as between patients with ADHD or other psychiatric disorders [35, 36, 39].

### Aim of the present study

In summary, there are several studies reporting promising results regarding the ability of the QbTest 6–12 and the Qb+^©^ to differentiate between patients with and without ADHD. Nevertheless, findings are inconsistent, and often suggest using neuropsychological tests only as an additional resource within a comprehensive assessment strategy incorporating a variety of methods [27]. In the clinical community there is a high controversy about the usefulness of objective measures for diagnostic purposes as problems regarding sensitivity, specificity and ecological validity have been reported [40]. In light of the evidence that ratings and tests seem to assess partly different constructs, objective and subjective measures could be seen as complementing each other. The diagnostic value of objective tests becomes all the more important when the potential risks of subjective measures are taken into account, informant bias being the most important thereof. There is lack of studies evaluating the differential contributions of objective and subjective measures for correctly classifying ADHD. Whilst the study investigates the relative contribution of subjective measures in a classifier, the diagnostic accuracy using objective measurements only is considered more generalisable due to the inherent inter-operator variability in subjective measures. In contrast to most previous studies using neuropsychological measures and ratings to differentiate between patients with ADHD and healthy controls, we used machine learning rather than discriminant function analysis or logistic regression analysis. The advantage of the former is that it is data-driven and less sensitive to outliers [41]. Furthermore, it is a multivariate approach, as it does not rely on summary scores, but considers every single item. The risk of losing information is therefore reduced [41]. More specifically, the present study used support vector machine (SVM). This machine-learning approach is known to be very robust and capable of translating well in studies using imaging data [42]. However, it has been predominantly implemented in studies using neuroimaging data to diagnostically classify clinical populations [43, 44] and not in studies using standard clinical assessments as recommended by the various ADHD diagnostic guidelines outlined above.

The first aim of this study was thus to investigate the accuracy of employing only variables from the objective measures to reveal their specific potential contribution free from the potential confound of subjective measures. We further aimed to investigate how objective measures are related to subjective measures by investigating how well we could discriminate between ADHD and controls when using the combination of these two types of measures. In contrast to previous research, we used a machine-learning technique (SVM) to analyze the data.

## Methods

### Participants and procedure

Thirty children with ADHD and thirty controls matched at group level according to age and gender were enrolled in the childhood ADHD prediction. Thirty-eight adults with ADHD and thirty-eight age and gender-matched controls were enrolled in the adulthood ADHD prediction resulting in a total sample of N = 136. All children and adults with ADHD were recruited through an ADHD outpatient clinic within the university. Control children were recruited through local schools and children who participated in the study were given a movie voucher. Control adults were recruited at the university and via advertisements; they also received movie vouchers for study participation. No established or suspected ADHD diagnosis, or family history of ADHD were allowed for the individuals in the control groups.

Clinically-referred children were included in the study if they met the DSM-IV [45] criteria for ADHD (either combined, predominantly inattentive or predominantly hyperactive/impulsive subtype) and had an IQ-score ≥ 80 (short version of the Wechsler Intelligence Scale for Children IV [46]: block-design, similarities, digit span, information and picture arrangement; [47]). The exclusion criteria were symptoms of inattention, hyperactivity or impulsivity due to other medical conditions such as hyperthyroidism, autism, epilepsy, brain disorders and any genetic or medical disorder associated with externalizing behavior. Comorbid disorders like oppositional defiance disorder (ODD) or conduct disorder (CD) did not constitute an exclusion criterion as they are prevalent in about 30–50% of the population [48]. Other comorbid disorders (e.g. learning disorders, anxiety or depression) also did not result in exclusion as long as ADHD was the primary diagnosis. Participants were allowed to take medication but were asked to stop taking it 2 days before the objective tests were applied. Similar inclusion and exclusion criteria applied to adults with the exception that IQ was not assessed. Adult patients were recruited from a specialized outpatient clinic whose standard diagnostic procedure does not include extensive testing of cognitive abilities. Instead, achieved schooling and current job position are gathered. According to that, none of the patients were estimated to score below IQ of 80 as all of them have at least completed middle school. ADHD diagnoses were based on a DSM-IV-oriented clinical interview conducted by an experienced clinician, as this is known to be a highly reliable method for making an ADHD diagnosis [49–51]. For the children, we conducted the Schedule for Affective Disorders and Schizophrenia for School-Age Children-Present and Lifetime Version interview (K-SADS-PL; [52]) with a parent. Its inter-rater reliability ranges from .93 to 1.0 [53]. The adult patients completed the Wender Reimherr Interview (WRI; [54]), which has inter-rater reliability ranging from .45–.95 [55]. Rating scales were completed at home and the objective tests were instructed by clinicians or research assistants.

In the child sample, the male-to-female ratio was 21/9 (ADHD) and 19/11 (controls). The ADHD group’s mean age was 8.9 years (SD = 1.4, range 7.0–11.0 years) and the controls’ 8.7 years (SD = 1.2, range 6.9–10.8 years). A percentage of 26.7 of the children diagnosed with ADHD were predominantly inattentive, 3.3% were predominantly hyperactive-impulsive and 60% fulfilled the diagnostic criteria for the combined subtype. Unfortunately, for 10% of the children the subtype information was not available. The adult ADHD group’s mean age was 35.1 years (SD = 11.7, range 19–63 years); and the controls’ 32.2 years (SD = 9.6, range 21–56 years). Both adult groups had a male-to-female ratio of 25/13. A percentage of 10.5 of the adults diagnosed with ADHD were predominantly inattentive, 2.6% were predominantly hyperactive-impulsive and 81.6% fulfilled the diagnostic criteria for the combined subtype. Unfortunately, for 5.3% of the adults the subtype information was not available.

Children with ADHD had a significantly lower IQ than the controls (t (58) = − 4.49, *p* < .001), a factor known to be typical of this population (e.g., [56, 57]). In their twin-study, Kuntsi et al. [58] found that the association between ADHD and lower IQ is based predominantly on genetic influences rather than environmental effects. Controlling for IQ would therefore not have affected the composition of our ADHD-population. We thus decided against controlling for IQ as a possible confound because that can bias classification results. The high mean IQ of our ADHD (M = 113.1; SD = 11.6) and control groups (M = 125.8; SD = 10.8) is most likely due to the high percentage of children from academic families in a small university town (80.000 inhabitants of which 27.000 are students and ~ 10.000 academics working at the university with a further ~ 10.000 working in related academic institutions). Considering IQ’s high rate of heritability (one that even rises with age [59]) and the additional role of the socio-economic status of those with a high IQ in particular [60], our sample’s IQ values are not that surprising. For details on demographics, please see Table 1. Furthermore, Table 3 shows correlations between IQ and the subjective and objective variables.

**Table 1:** Demographic characteristics of the sample

	Adults		Children	
	ADHD	Controls	ADHD	Controls
n	38	38	30	30
Mean age (SD)	35.1 (11.7)	32.2 (9.6)	8.9 (1.4)	8.7 (1.2)
Gender				
Male (%)	25 (65.8)	29 (65.8)	21 (70)	19 (63.3)
Female (%)	13 (34.2)	16 (34.2)	9 (30)	11 (36.7)
Mean IQ (SD)	N/A	N/A	113.1 (11.6)	125.8 (10.8)

*SD* standard deviation, *N/A* not available

### Measures

The standard diagnostic procedure for ADHD at the outpatient clinic from which our participants were recruited incorporates a variety of measures like clinical interviews, self- and observer ratings and neuropsychological tests. Instruments used for our SVM analyses are described in greater detail below.

#### Conners ADHD rating scales self- and observer rating long version (CAARS-L: S/O)

The CAARS-L: S [61] is a self-rating instrument that assesses ADHD symptoms in adults aged 18 years and above. The long version consists of 66 items rated on a 4-point Likert-type scale ranging from 0 (*not at all/never)* to 3 (*very much*/*very frequently*). Factor analyses for the original and the German version have supported a four-factor structure consisting of inattention/memory problems, hyperactivity/restlessness, impulsivity/emotional lability, and problems with self-concept [62, 63]. The internal consistency of each of these subscales ranges between .82 and .85, and all four subscales reveal high sensitivity and specificity [63]. The CAARS-L: S thus represents a reliable and cross-culturally valid measure of current ADHD symptoms in adults [64]. The observer version CAARS-L: O also assesses ADHD symptoms using the same items and rating scale as the CAARS-L: S, but symptoms are rated by someone who has a close relationship with the subject under examination [65]. Observer ratings were performed by a person with a close relationship to the participant. In most cases, their partner or spouse was selected but it was sometimes a parent or close friend. This version’s factorial validity has been confirmed and its psychometric properties proved to be satisfactory. In our study the internal consistency of the CAARS-L: S/O ranged from α = .90/.89 to α = .95/.94.

#### Conners-3 parent/teacher ratings

The Conners-3 parent/teacher ratings [66] are two questionnaires assessing ADHD symptoms and associated problems like oppositional behavior or learning problems in children and adolescents aged 6–18 years. The long version contains 105/111 items (parent/teacher) rated on a 4-point Likert-scale from 0 (*not at all*/*never*) to 3 (*very much*/*very frequently*). The Conners 3 incorporates the following ten scales: inattention, hyperactivity/impulsivity, learning problems, executive functions, aggression, peer relations (content scales); DSM IV-inattention, DSM IV-hyperactivity/impulsivity, DSM IV-conduct disorder, DSM IV-oppositional defiant disorder (symptom scales); ADHD index, Global index. The German version has revealed good to very good internal consistency (Cronbach’s α = .74–.96). Also, confirmatory factor analyses of the Conners 3 German version confirmed the factor structure for the content scales in the original American version [67]. In our sample we found a Cronbach’s alpha of .85 for the content scales and α = .79 for the symptom scales of the Conners 3 parent rating scale.

#### Quantified Behavior Test for adolescents and adults (Qb+^©^)

The Qb+^©^ [30] is a continuous performance task (CPT) measuring sustained attention combined with a simultaneous high resolution motion tracking system that takes 20 min to complete. Presented stimuli are a blue circle, a blue square, a red circle, and a red square. A response key is to be pressed when two identical stimuli are shown in succession. The target/non-target ratio is 25/75. During performance, the participant’s head movements are recorded with an infrared camera tracking a reflective marker attached to the headband the participant is wearing. One thus obtains data from a total of nine parameters that measure each of the three core ADHD symptoms. Activity is measured by five parameters: time active (i.e., time the subject has moved more than 1 cm/s in percentage of the task’s entire duration), Distance (i.e., distance traveled by the reflective headband marker in m), area (i.e., surface covered by the headband reflector during the test in cm^2^), microevents (i.e., small movements exceeding 1 mm), and motion simplicity (i.e., complexity of the motion pattern in  %). Inattention is measured by the following parameters: reaction time (RT), RT variability (i.e., standard deviation of RT in ms), and omission errors. The third ADHD domain, impulsivity, is assessed by commission errors. Psychometric properties of the Qb+^©^ are described in the introduction.

#### Quantified Behavior Test for children aged 6–12 (QbTest)

Similar to the Qb+^©^, the QbTest [29] consists of a standard CPT and a parallel registration of the participants’ movements with an infrared camera following a reflective marker attached to a headband. Stimuli are either a gray circle (target) or a gray circle with a cross (non-target) in random order of appearance. These are presented for 100 ms at an inter-stimuli-interval of 1900 ms. The target/non-target ratio is 50/50. The task is to press a button as quickly as possible when the target appears. The same parameters measuring the three core ADHD symptoms in the Qb+^©^ are also included in the childhood version (see descriptions above). In addition to these nine parameters, inattention also includes normalized variability in reaction time (i.e., RTVar divided by RT) and the impulsivity factor contains anticipatory reactions (i.e., reactions < 150 ms are considered coincidental). The QbTest’s psychometric properties are described in the introduction.

#### Test battery of attention for adolescents and adults and for children aged 6–11 (TAP/KiTAP)

The TAP’s [24] and KiTAP’s [25] three subtests below were used for our assessment of both age groups: Go/NoGo, divided attention and sustained attention. The Go/NoGo task assesses selective attention and in it, participants are instructed to press a button (“go”) when a target stimulus appears. For example, in the TAP an “×” and a “+” are presented and participants have to press the button when the “×” appears, but not when the “+” appears. In the divided-attention subtest, a visual and auditory task have to be processed simultaneously. In the sustained attention task, one has to be attentive for a period of about 15 min. The KiTAP’s tasks are embedded in a story about a haunted castle. In the divided-attention task, children look at an owl that shuts its eyes from time to time while they hear a low or high sound. Their task is to press the button either when the owl shuts its eyes or when they hear the same sound twice in a row. In the sustained-attention task on the other hand, ghosts in different colors are presented and children have to press a button if the same-colored ghost appears twice in a row. Psychometric properties are described in the introduction.

### Analyses

Analyses apply feature selection to identify those variables most relevant to the diagnosis, and we took a popular pattern recognition approach (SVM) to make the diagnosis prediction. To ensure each prediction was made based on data that was novel to the classifier, as would occur in routine clinical practice, we used cross-validation. Due to the differences in measures for childhood and adulthood ADHD diagnoses, we carried out two separate but identical analyses for each age group.

#### Variable preparation

As the first step, the variables were standardized to reduce errors due to scaling. This involved subtracting the mean value of each variable and dividing by the standard deviation. Standardization aims to ensure that the automated selection of variables is based on their predictive value, rather than on their relative variability or magnitude.

#### Individual scan classification

All analyses were performed in Matlab 2012a (The Mathworks Inc.) and Matlab-based calculations used the SVM toolbox [68] and custom Matlab scripts. To investigate which variables predicted ADHD diagnosis, we applied a linear support vector machine (SVM; [69, 70]) pattern-recognition method to each dataset, with standard leave-one-out cross validation (LOOCV). The advantages and technical details of SVM and pattern-recognition approaches in general are described in more detail elsewhere [43, 71]. Put simply, one subject is removed from the data set and the aim is to identify the set of variables that best separate the N-1 subjects into patients and controls. The optimal set of variables is then used to predict whether the subject that was removed belongs to the patient or control group. This process is repeated until all subjects have been classified.

To identify which variables are most important to the prediction we employed feature selection. This technique involves ranking the variables from largest to smallest absolute differences between groups within each training set (excluding the subject left out to ensure it is novel to the classifier). Potential thresholds were explored over a wide range, whereby all variables with differences between the groups above the threshold are included in the classification. The threshold that yielded the highest training stage accuracy (the accuracy obtained during the second [inner] LOOCV procedure—which does not include the novel data) was used in the final prediction. This approach has been described in greater detail elsewhere [72]. Notably, as feature selection took place for each training set (each combination of N-1 subjects), a different combination of variables can be selected for each subject’s prediction. This approach optimized the number of variables required to classify the data. We calculated the classification accuracy, sensitivity, and specificity at each stage. In addition to the approach including all variables, we investigated the feasibility of predicting applying the objective QbTest and TAP scores only (versions for both children and adults) to see whether they could independently predict diagnosis without relying on the subjective Conners’ scores.

## Results

Our child datasets included age, gender, Conners-3 parent/teacher ratings, QbTest 6–12 and the KiTAP scores; the adult datasets age, gender, CAARS-L: S/O, QbTest+© and TAP. IQ and medication history were not included in the prediction for reasons as outlined above. Tables 2 and 3 show correlations between age, IQ (child sample), the symptom scales of the subjective measures and objective variables.

**Table 2:** Correlations between age, the symptom scales of the CAARS: S/O, variables of the TAP and the Qb+^©^ factors for the adult sample

	1	2	3	4	5	6	7	8	9	10	11	12	13	14	15	16	17	18	19	20
1 age	–																			
2 CAARS: S DSM IA	.04	–																		
3 CAARS: S DSM HY/Imp	.16	.03	–																	
4 CAARS: S DSM ADHD	.16	.50**	.88**	–																
5 CAARS: O DSM IA	− .02	.41**	.10	.29*	–															
6 CAARS: O DSM HY/Imp	.16	.02	.53**	.47**	− .00	–														
7 CAARS: O DSM ADHD	.16	.12	.54**	.52**	.24*	.97**	–													
8 TAP1 reaction time	.22	.21	.14	.22	.17	− .06	− .05	–												
9 TAP1 RT SD	− .08	.15	.09	.15	.33**	− .13	− .05	.43**	–											
10 TAP1 errors	− .21	.05	− .04	− .02	.18	.15	.19	− .28*	.13	–										
11 TAP1 omission errors	− .19	.09	.12	.15	.24*	.10	.15	.14	.43**	.12	–									
12 TAP2 errors	− .08	− .13	.04	− .03	.14	.06	.09	.15	.36**	.21	− .01	–								
13 TAP2 omission errors	.09	.25*	.14	.24*	.34**	.07	.15	.25*	.19	.11	.25*	.10	–							
14 TAP3 reaction time	.33**	.10	.02	.06	.18	.03	.07	.55**	.25*	− .03	.06	.10	.17	–						
15 TAP3 RT SD	.09	.03	− .01	.01	.13	.03.	.06	.11	.34**	.03	.15	.05	.12	.43**	–					
16 TAP3 errors	− .03	− .02	.19	.16	.13	− .03	.01	.00	.13	.18	.27*	.22	.09	− .15	.05	–				
17 TAP3 omission errors	.10	.29*	.18	.30**	.48**	.06	.18	.20	.28*	.12	.35**	.11	.39**	.41**	.33**	.26*	–			
18 Qb activity	− .02	.20	.18	.25*	.38**	− .07	.02	.26*	.29*	.06	.21	.17	.10	.33**	.16	.15	.43**	–		
19 Qb impulsivity	.13	.12	.01	.07	.26*	.15	.21	− .09	.13	.36**	.39**	.22	.38**	− .06	.15	.51**	.37**	.05	–	
20 Qb inattention	.26*	.14	.11	.17	.32**	.07	.15	.50**	.29*	.19	.11	.29*	.20	.65**	.39**	.11	.53**	.45**	.13	–

*CAARS: S* CAARS self rating, *CAARS: O* CAARS observer rating, *IA* inattention, *HY/Imp* hyperactivity/impulsivity, *TAP1* Go/Nogo task of the test battery of attention, *TAP2* selective attention task, *TAP3* sustained attention task, *RT* reaction time in ms, *SD* standard deviation

* p < .05 level (two-tailed) ** p < .01 level (two-tailed)

**Table 3:** Correlations between age, IQ, the symptom scales of the Conners-3 parent ratings, variables of the KiTAP and the QbTest factors for the child sample

	1	2	3	4	5	6	7	8	9	10	11	12	13	14	15	16	17	18	19	20
1 age	–																			
2 IQ	.01	–																		
3 CP DSM inattention	− .15	− .41**	–																	
4 CP DSM HY/Imp	− .31*	− .18	.70**	–																
5 CP ADHD index	− .27*	− .38*	.89**	.82**	–															
6 KiTAP1 RT	− .21	− .10	.19	.21	.22	–														
7 KiTAP1 RT SD	− .02	− .23	.04	.09	.13	.44**	–													
8 KiTAP1errors	− .11	− .01	− .21	− .21	− .20	− .40**	.04	–												
9 KiTAP1omission errors	− .02	.11	− .14	− .04	− .17	.01	.12	.14	–											
10 KiTAP2 RT	− .29*	− .15	.24	.22	.25	.53**	.23	− .01	− .11	–										
11 KiTAP2 RT SD	− .11	− .09	− .07	− .12	− .02	− .03	.20	.20	− .06	.14	–									
12 KiTAP2 errors	− .16	− .25	− .04	− .06	− .07	− .09	.25	.26*	− .05	.09	.32*	–								
13 KiTAP2 omission errors	− .18	− .18	.13	.04	.15	.02	.15	− .01	− .05	.07	.39*	.54**	–							
14 KiTAP3 RT	− .33*	− .10	− .09	.03	.05	.58**	.32*	− .11	.03	.50**	.06	.09	.17	–						
15 KiTAP3 RT SD	− .15	− .27*	.05	.04	.10	.35**	.32*	− .17	.04	.26*	.10	.17	.14	.44**	–					
16 KiTAP3 errors	− .00	− .11	.23	.18	.22	− .01	.21	.30*	− .04	− .09	.01	.13	.06	− .01	.30*	–				
17 KiTAP3 omission errors	− .26*	− .26*	.19	.20	.35**	.21	.51**	.15	.03	.23	.27*	.30*	.37**	.36**	.44**	.26*	–			
18 Qb activity	.03	− .33*	− .02	.05	.01	.00	.04	.05	.11	.03	− .12	.06	.12	.20	.14	.23	− .04	–		
19 Qb impulsivity	.01	− .30*	.15	.08	.21	− .26*	.11	.37**	− .09	− .10	.36**	.18	.10	− .20	− .00	.47**	.01	.18	–	
20 Qb inattention	.27*	− .35**	.13	− .03	.12	.17	.23	− .02	.14	.25	.13	− .05	.19	.31*	.27*	.25*	.23	.41**	.22	–

*CP* Conners’ parents scale, *HY/Imp* hyperactivity/impulsivity, *KiTAP1* Go/Nogo task of the test battery of attention for children, *KiTAP2* selective attention task, *KiTAP3* sustained attention task, *RT* reaction time in ms, *SD* standard deviation

* p < .05 level (two-tailed) ** p < .01 level (two-tailed)

As expected, we found significant negative correlations between IQ, the Conners’ symptom scale for inattention and the ADHD index as well as the Qb factors. Furthermore, there were significant positive correlations between corresponding variables of the different TAP/KiTAP tasks. Regarding the adult sample, the Qb factor for impulsivity correlated significantly positive with the number of errors of most of the TAP tasks, indicating that patients who produced a lot of errors in the TAP also scored high on the impulsivity factor of the Qb+^©^. Additionally, the Qb factor inattention correlated significantly positive with the inattention symptom scale of the CAARS observer scale as well as with the TAP variables reaction time and reaction time variance (RT SD). Finally, there were significant correlations between the number of omission errors in the TAP tasks and the ADHD and inattention symptom scales of the CAARS. Regarding the child sample, we also found positive correlations between the Qb impulsivity factor and the number of errors in the KiTAP tasks Go/Nogo and sustained attention. Furthermore, there were positive correlations between the Qb inattention factor and the reaction time and reaction time variance of the sustained attention task. In general, there were only few significant correlations between the subjective and the objective variables, a fact already shown in previous studies [31, 37].

### Prediction of ADHD diagnosis in adults

Using a linear SVM and feature selection on the adult dataset, we were able to predict an ADHD diagnosis with 89.5% accuracy (sensitivity = .90, specificity = .90, χ^2^ = 44.26, p < .0001). The majority of the variables relevant to the classification were selected from the CAARS scores. Many of the scores from the self-ratings were especially predictive, with each of the 51 variables selected in 36.20% of the predictions on average. Similarly, the observer-rated scores contained many predictive variables, with each variable selected in 20% of the cross-validated classifiers on average. This was expected, as symptom severity scores are likely to readily distinguish patients from controls.

We did not find the test battery of attention scores to be particularly predictive of ADHD diagnosis. Of the 28 variables included in the calculation, each variable was selected in 2.96% of the predictions on average. The 16 QbTest+© variables entered into the classifier were selected in 8.96% of the predictions on average.

The variables selected in all of the predictions were questions from the self- and observer-rated CAARS. From the self-ratings, we found the following items to be relevant to all predictions: “*I have trouble keeping my attention focused when working*”, “*I feel restless inside even if I am sitting still*”, “*Things I hear or see distract me from what I’m doing*”, and “*I am restless or overactive*”. Similarly, three items from the observer-rated questionnaire were used in all predictions: “*is easily frustrated*”, “*is distracted by sights or sounds when trying to concentrate*”, and “*can’t keep his/her mind on something unless it’s really interesting*”.

In addition, although not used in all the predictions, a number of self-rated CAARS items were selected in over 75% of predictions: “*Many things set me off easily*”, “*My moods are unpredictable*”, “*Sometimes my attention narrows so much that I’m oblivious to everything else; other times it’s so broad that everything distracts me*”, “*I can’t keep my mind on something unless it’s really interesting*”, “*I am distracted when things are going on around me*”.

When including only objective measures (the output from the QbTest+© and TAP tasks), we predicted an ADHD diagnosis with 79% accuracy (sensitivity = .82, specificity = .76, χ^2^ = 23.28, p < .0001). The following variables were used in over 85% of predictions: overall omission errors made at the subtest sustained attention in the TAP; QbTest+©: omission errors, error rate, normalized reaction time variance, normalized reaction time variance without outliers, and the ability of the patient to distinguish between target and non-target.

### Prediction of ADHD diagnosis in children

By applying the same technique used to predict diagnosis in the adult population to 30 children with ADHD and 30 controls, we were able to predict diagnosis with 86.7% accuracy (sensitivity = .83, specificity = .90, χ^2^ = 29.53, p < .0001). As in the adult study, the Conners’ scores proved to be the most predictive of an ADHD diagnosis. Only 12 parent-rated scores were entered into the classification procedure, and those variables were selected in 36.53% of the predictions on average. The Conners’ parent subscores relating to executive function, inattention DSM-IV ratings and the ADHD index were selected in all predictions, and the general inattention score was selected in 75% of the predictions. None of the variables taken from the KiTAP were selected in any of the predictions, while the 15 QbTest 6–12 variables entered into the classifier were selected in only 1.11% of the predictions on average.

When using only the output from the QbTest 6–12 and KiTAP tasks, it was possible to predict an ADHD diagnosis with 78% accuracy (sensitivity = .80, specificity = .77, χ^2^ = 17.09, p < .0001). The following variables were used in over 98% of predictions: KiTAP: sustained attention—number of omission errors, GoNogo—median of reaction time and number of errors; QbTest 6–12: distance participant moved during testing, area covered by the patient during testing, micro movements exceeding 1 mm, complexity of movement-pattern, multiple pressing of the test button, reaction time variance without outliers, normalized reaction time variance without outliers, anticipatory, reactions < 150 ms that are considered accidental. All prediction results are summarized in Table 4.

**Table 4:** Prediction results

	Adult prediction		Child prediction	
	All variables	Objective variables	All variables	Objective variables
Accuracy (%)	89.5	79.0	86.7	78.0
Sensitivity	.90	.82	.83	.80
Specificity	.90	.76	.90	.77

## Discussion

In this study we aimed to determine the accuracy with which objective and subjective measures can predict the diagnosis in individuals with and without ADHD. Based on the fact that questionnaire data suffer from rater bias and that diverse methods seem to assess partly different constructs, we wanted to examine both the combined effect of subjective and objective measures as well as the unique effects of the objective measures obtained from the QbTest 6–12, Qb+^©^, KiTAP or TAP.

### ADHD assessment in adults

Our results demonstrate that using both subjective and objective measures in adults, it was possible to predict an ADHD diagnosis with an accuracy of 89.5%, with self-rated scores being the most predictive ones (selected in an average of 36.20% predictions) followed by the observer-rated scores (selected on average in 20%). Variables of both the Qb+^©^ and TAP were less relevant to predicting an ADHD diagnosis than were the self- and observer-ratings. Nevertheless, prediction was still possible using only these objective measures with an accuracy of 79%.

Regarding the TAP, the variable omission errors from the subtest “sustained attention” was selected in more than 85% of the predictions; the same accounts for the Qb+^©^ variables omission errors, error rate, reaction time variance, and the participant’s ability to distinguish between target and non-target. This finding is in line with previous findings, as a review of 33 studies on the neuropsychology of adults with ADHD identified omission errors and reaction time variance as variables well able to discriminate between adults with ADHD and controls [73]. This is further supported by our analysis that revealed some significant correlations between the number of omission errors in the TAP, the overall ADHD and inattention symptom scales of the CAARS. These two variables are generally considered to be indicative of inattention, which has been supported in previous factor analyses [38]. Our findings thus reveal those variables indicative of inattention as the most predictive ones for diagnosing ADHD in adults. This is in line with research suggesting that inattention is the ADHD symptom domain most likely to persist through lifetime, while hyperactivity and impulsivity seem to decline to a greater extent [74]. It is unlikely that these results are due to our sample having a higher proportion of the inattentive subtype as those were only 10.5%, but 81.6% of the participants had the combined subtype. Furthermore, our findings demonstrate high accuracy rates when the prediction was based on objective measures only, thus supporting their diagnostic value independently of subjective measures, removing a significant source of variability and thus increase the likelihood that the classification accuracy obtained would be able to be independently replicated. This is important, as in clinical practice we do not just want to know whether patients are inattentive in daily life (due to several potential reasons besides a primary attention deficit), but also whether they are inattentive in structured situations such as when completing a neuropsychological task. Thus, as emphasized by Toplak et al. [23], ratings and tests capture at least partly different constructs and should therefore be seen as complementing one another. This is further supported by our findings that showed rather low and non-significant correlations between subjective and objective measures. Furthermore, an objective assessment of a patient’s impairment might be especially relevant in cases where observer-ratings are unavailable [75], when self-ratings might be questionable due to the potential faking of results (e.g., [76, 77]), or when answers are considered biased [18]. Recent studies have provided additional support for the value of objective assessments. For example, Hirsch and Christiansen [78] showed that inattention as measured with the Qb+^©^ was indicative of overall impairment, adding key supplemental information. Another study demonstrated that the Qb+^©^ is sensitive to medication effects, showing an improvement in 54% of patients who reported no changes in the subjective measure [79].

### ADHD assessment in children

We were able to predict an ADHD diagnosis in children based on all of the variables with 86.7% accuracy. Here, no self-ratings were used, but the sub-scores “executive functioning”, “DSM IV inattention” and the “ADHD index” of the parent-ratings were selected in all predictions demonstrating their diagnostic value. The prediction using the objective measures did not produce as accurate a classifier as the combined objective and subjective measures in our child sample. However, predicting an ADHD diagnosis using only the objective measures was still significant with 78% accuracy.

Similar to the adult sample, the variable omission errors of the subtest “sustained attention” of the KiTAP was selected in more than 98% of the predictions, as was the median of the reaction time and number of errors in the “Go/NoGo” subtest. This is in line with previous research showing that children with ADHD commit both more omission errors and commission errors than healthy controls [80–82]. Looking at the QbTest 6–12, the most important variables for the prediction differ from those of the adult sample, as the variables assessing hyperactivity were predominantly selected, namely the distance moved and the area covered by the participant, micro-movements, complexity of movement patterns, as well as multiple pressing of the button, reaction time variance, and reaction times < 150 ms. This reflects the findings on the development of ADHD symptoms from childhood to adulthood, demonstrating that hyperactivity is more observable in children than in adults [14, 83, 84]. Adults, in contrast, report more feelings of restlessness or being driven by an internal motor than overtly exhibiting hyperactive behavior. Furthermore, it is interesting that for the QbTest, the variables assessing hyperactivity were most often selected for the prediction, whereas for the Conners-3 parent ratings the sub-score assessing inattention seemed to be more important. Hyperactive behavior thus appears to be evaluated better applying objective measures rather than subjective reports. A likely reason for this finding is that the QbTest can capture tiny movements that the patient alone might not even notice. Including objective measures thus resulted in high accuracy for the children. Regarding hyperactivity, the objective measures even outperformed the ratings. These findings support the inclusion of objective measures in ADHD diagnostics.

We found significant negative correlations between IQ, the Conners’ symptoms scale of inattention and the ADHD index, two of the KiTAP variables indicative for inattention (reaction time variance and omission errors) and the Qb factors. This is not surprising as the children with ADHD had lower IQ, which is known to be typical for this population [56, 57]. The fact that we found these correlations not only for the objective measures but also for some of the subjective variables underlines that the performance in the objective tasks was not only due to the lower IQ, but also influenced by the deficits these children present.

### Limitations and future research

According to the DSM-criteria [1] for ADHD, impairment should be established in multiple settings. It would therefore have been valuable to also include teacher ratings in the present study. Unfortunately, there was too much missing data for the teacher ratings to be absorbed in our analyses. This is a problem we confront constantly during our daily diagnostic routine. Here, objective measures are given additional weight, as they have the potential to add valuable information not otherwise obtainable.

Another limitation of our study is the relatively small number of participants in each group (adults ADHD/controls: 38; children ADHD/controls: 30). As we aimed to be able to make reliable claims regarding the diagnostic accuracy of the instruments assessed, we focused on having gender- and age-matched groups. This resulted in relatively small group sizes, but the total number of participants (N = 136) can be regarded as satisfactory.

Future research should preferably include a clinical control sample, as it is highly relevant for clinicians to be able to distinguish between patients with ADHD and those exhibiting similar symptoms due to another underlying disorder (e.g., inattention is also common in patients with depression).

## Conclusions

In conclusion, we took a sophisticated statistical approach in this study to examine the diagnostic contributions of various measures assessing ADHD and to assess their classification accuracy. The present findings are highly relevant for clinicians, and can help to improve the workup for diagnosing ADHD. Our investigation’s findings demonstrate that when using both subjective and objective measures, an ADHD diagnosis can be accurately predicted with high sensitivity (adults: .90, children and adolescents: .83) and specificity (adults: .90, children and adolescents: .90). Whilst the combination of objective and subjective measures produced more accurate results than the classification based on objective measures only, the latter was still highly satisfactory and removes a potential source of error. The core symptom of hyperactivity is captured especially well in children via objective measures. Considering the evidence that ratings and tests seem to assess at least partly different constructs [23], objective measures always add unique information. Considering that our study revealed only objective measures to be highly accurate, the fact that subjective measures are always influenced by rater bias, and that teachers’ appraisals are not made routinely available to clinicians, we recommend that objective measures be included in ADHD diagnostics not only to supplement ratings, but as an integral element thereof.
